# Interpretable Machine Learning Prognostic Model Integrating Inflammatory-Nutritional Biomarkers for Post-Prostatectomy Biochemical Recurrence: Development, Internal Validation, and Translational Target Exploration

**DOI:** 10.3390/curroncol33100608

**Published:** 2026-10-09

**Authors:** Hao Wang, Yifei Zhang, Zhen Li, Jiyue Wu, Huawei Cao, Lijian Gan, Xuemeng Qiu, Wei Wang

**Affiliations:** Department of Urology, Beijing Chao-Yang Hospital, Capital Medical University, Beijing 100020, China

**Keywords:** prostate cancer, biochemical recurrence, machine learning, lymphocyte-to-monocyte ratio, prognostic nutritional index, SHAP, molecular docking

## Abstract

**Objective**: Biochemical recurrence (BCR) after laparoscopic radical prostatectomy (LRP) significantly affects the long-term survival of prostate cancer (PCa) patients. This study aimed to develop and validate an interpretable machine learning model integrating clinicopathological characteristics and preoperative inflammatory and nutritional indices to predict recurrence-free survival (RFS) and explore potential molecular therapeutic targets. **Methods**: A retrospective cohort of 320 PCa patients undergoing LRP at Beijing Chaoyang Hospital, Capital Medical University was analyzed. We integrated demographic, clinicopathological variables, and seven composite inflammatory/nutritional indices before LRP. A Mime-based machine learning survival pipeline was employed, combining Cox-based feature selection with survival algorithms. Model performance was evaluated using the concordance index (C-index), time-dependent ROC, calibration curves, and decision curve analysis (DCA). SHAP (SHapley Additive exPlanations) was used for model interpretation and deriving clinical cutoffs. Furthermore, intersecting genes of BCR, lymphocyte-to-monocyte ratio (LMR), and prognostic nutritional index (PNI) were identified via GeneCards, followed by drug sensitivity screening and molecular docking. **Results**: The StepCox[both] combined with Random Survival Forest model demonstrated optimal performance, achieving a validation C-index of 0.788. SHAP analysis identified the top five predictive factors for recurrence: preoperative LMR, PNI score, postoperative Gleason score ≥ 8, positive surgical margin, and lymphovascular invasion. Model-derived cutoffs were determined as LMR = 3.9 and PNI = 48.8. Intersection analysis revealed 1211 shared targets among BCR, LMR, and PNI, with TP53 and PTEN ranking highest. Drug sensitivity analysis and molecular docking suggested potential inhibitory effects of compounds like sabutoclax and dordaviprone on these targets. **Conclusions**: The proposed model may serve as a robust and interpretable risk stratification for postoperative PCa patients. The integration of clinical phenotyping with exploratory molecular docking offers a translational pathway from prognostic indices to potential therapeutic hypotheses, warranting further prospective validation.

## Simple Summary

Prostate cancer is one of the most common cancers in men, and disease recurrence after radical prostatectomy remains a key clinical challenge. Current risk assessment approaches rely mostly on tumor pathology features and may not fully capture individual differences in disease progression. This study developed an interpretable machine learning model combining routine clinical data, tumor pathology, and blood markers reflecting preoperative inflammation and nutritional status. The study also explored potential molecular targets and candidate therapeutic compounds linked to recurrence risk. These preliminary findings may help inform personalized postoperative care strategies and support future translational research.

## 1. Introduction

Prostate cancer (PCa) remains one of the most prevalent malignancies and a leading cause of cancer-related mortality among men globally [1]. Laparoscopic radical prostatectomy (LRP) is a standard curative treatment for localized PCa, yet postoperative biochemical recurrence (BCR) occurs in a substantial proportion of patients and serves as a critical early marker of disease progression [2,3]. Accurate postoperative risk stratification is essential for guiding adjuvant therapy decisions, optimizing follow-up schedules, and ultimately improving recurrence-free survival (RFS). Conventional prognostic tools, including the D’Amico risk classification, Gleason score, and prostate-specific antigen (PSA) level, provide only moderate discriminatory performance and fail to fully capture the heterogeneous biological features of individual tumors, leaving an unmet clinical need for more precise, practical, and biologically informed prediction models [4,5].

Chronic inflammation and nutritional impairment are well-recognized hallmarks of cancer progression, exerting profound effects on tumor proliferation, invasion, and treatment response in PCa [6,7]. In recent years, composite inflammatory and nutritional indices derived from routine blood and biochemical tests, such as the prognostic nutritional index (PNI), lymphocyte-to-monocyte ratio (LMR), and pan-immune-inflammation value (PIV), have emerged as promising, cost-effective prognostic biomarkers [8,9]. PNI is a non-invasive biomarker calculated from serum albumin and peripheral blood lymphocyte counts, and it can reflect cancer patients’ nutritional status and systemic inflammatory burden. Meanwhile, a reduced LMR essentially reflects a state of suppressed anti-tumor immunity and a dominant pro-tumor microenvironment. To date, accumulating meta-analyses have demonstrated that both PNI and LMR exhibit significant prognostic value in patients with PCa [10,11,12]. Despite these advances, current PCa prognostic research still faces two key unresolved limitations. Most studies have focused on the prognostic value of individual composite markers rather than systematically integrating multiple indices alongside conventional clinicopathological factors. In addition, machine learning-based prognostic models exhibit limited interpretability and lack well-validated cutoff values, which greatly hinder their translation into routine clinical practice [13].

Beyond suboptimal prognostic stratification performance, another critical research gap lies in the disconnect between clinical prognostic indices and their underlying molecular mechanisms. Most prediction models focus primarily on risk discrimination, with limited subsequent exploration of the core drivers, actionable molecular targets, and the therapeutic implications that may be concealed within these validated peripheral prognostic signatures. With the rapid development of bioinformatics databases and computational structural biology approaches, an integrated translational analytical workflow has become technically accessible to connect clinical phenotypic markers with tumor molecular landscapes [14,15,16]. It has become feasible to identify disease-related targets and screen candidate agents based on clinical indices, providing a rational path from clinical risk prediction to therapeutic hypothesis generation.

Accordingly, the present study was designed to develop and validate an interpretable RFS prediction model for patients with PCa undergoing LRP. This model integrated demographic characteristics, conventional clinicopathological parameters, and multiple composite inflammatory and nutritional indices. We employed a Mime-based machine learning survival pipeline, combining multiple Cox-based feature selection strategies with established survival algorithms. The model’s performance was comprehensively evaluated using various metrics assessing discrimination, calibration, risk stratification capacity, and clinical utility. Survival SHAP analysis was performed to interpret model behavior and derive exploratory cutoffs for key composite indices. Furthermore, we performed target identification, drug sensitivity screening, and molecular docking analyses to identify shared molecular targets and candidate therapeutic agents linked to both BCR and the core prognostic indices. This study aims to provide a potentially reliable, interpretable tool for postoperative risk stratification of PCa and to supply exploratory molecular evidence for subsequent mechanistic investigations and targeted therapy development.

## 2. Methods

### 2.1. Study Design and Outcomes

This retrospective study analyzed patients with PCa who underwent LRP at Beijing Chaoyang Hospital, Capital Medical University, between January 2017 and December 2023. The inclusion criteria were as follows: (1) diagnosis of PCa confirmed by preoperative prostate biopsy and postoperative pathological examination; (2) no history of preoperative radiotherapy or endocrine therapy; (3) complete medical records, including routine blood and biochemical laboratory results obtained within one week preoperatively, as well as relatively comprehensive follow-up data; (4) no hematological or immune system disorders, and no active infections. The major exclusion criteria comprised unresectable tumors, concurrent malignant neoplasms, and surgical contraindications.

After LRP, all patients were divided into the non-recurrence group and the recurrence group. Patients with BCR were assigned to the recurrence group. BCR was specifically defined as two consecutive PSA values exceeding 0.2 ng/mL after LRP [17]. The first postoperative PSA was assessed at 4–6 weeks after surgery. For the first 2 years after surgery, PSA was monitored every 3 months; thereafter, PSA testing was performed every 6 months. Patients with postoperative PSA persistence were excluded; only patients whose PSA initially declined to an undetectable level and subsequently rose to meet the BCR criteria were included. RFS was defined as the time from surgery to BCR or to December 31, 2024, for patients without recurrence. During follow-up, when PSA rose above 0.2 ng/mL, patients underwent further imaging examinations including contrast-enhanced CT and whole-body bone scan. This study was performed in compliance with the Declaration of Helsinki and approved by the Ethics Committee of Beijing Chaoyang Hospital (Approval No.: 2020-science-299-1).

### 2.2. Study Variables and Analytical Workflow

Candidate variables comprised demographic variables, clinicopathological variables, and composite inflammatory or nutritional indicators. Pathological lymph node status was recorded as pN0, pN1, or pNx. Patients classified as pNx did not undergo pelvic lymph node dissection (PLND). PLND was routinely recommended for patients with intermediate- or high-risk PCa based on preoperative clinical risk stratification, while PLND was omitted for selected patients with low risk or significant comorbidities after multidisciplinary discussion.

The final prediction model incorporated age, body mass index (BMI), D’Amico risk classification, postoperative Gleason score, surgical margin status, perineural invasion, lymphovascular invasion, pathological lymph node status, T stage, LDH-to-albumin ratio (LAR), positive core density (PCD), lymphocyte-based prostate-specific antigen density (LB-PSAD), PIV, hemoglobin-to-platelet ratio (HPR), PNI score, PSA-PNI and LMR. Composite indices were calculated as follows: LAR = Lactate Dehydrogenase/Albumin [18]; PCD = Number of positive biopsy cores/Prostate volume [19]; LB-PSAD = PSAD/Lymphocyte count; PIV = (Neutrophil count × Platelet count × Monocyte count)/Lymphocyte count [20]; HPR = Hemoglobin/Platelets [21]; PNI = Serum Albumin + 5 × Total Lymphocyte count [22]; PSA-PNI = PSA × PNI; LMR = Lymphocytes/Monocytes [23].

The analytical workflow consisted of three sequential stages. First, clinical variables and composite indices were screened for intergroup differences, collinearity, and logistic associations with BCR. Second, survival models were developed and interpreted to identify predictors of RFS. Third, the two most stable composite indices identified from survival analyses were subjected to GeneCards target screening and exploratory drug response analyses. This staged framework logically linked clinical prediction and target discovery while distinguishing their respective levels of evidence.

All analyses were designed to address three distinct objectives: risk prediction, model interpretation, and biological hypothesis generation. Logistic regression and survival models were used to evaluate variable associations and RFS risk within the clinical cohort. SHAP analyses were further performed to interpret model performance and determine model-derived cutoff values, with no causal inference drawn in this procedure. Finally, analyses based on the GeneCards and CellMiner databases as well as molecular docking were utilized to generate target and drug hypotheses based on clinical prognostic indices. Such hierarchical analysis was critical because each analytical layer delivered a distinct degree of inference: cohort-level risk prediction, model-based interpretation, database-assisted target prioritization, and in silico verification of molecular structural feasibility.

### 2.3. Descriptive Statistics and Group Comparisons

Continuous variables were summarized as mean ± standard deviation when approximately normally distributed and as median with interquartile range otherwise. Categorical variables were summarized as counts and percentages. Between-group comparisons used Student’s t-test or the Mann–Whitney U test for continuous variables and the chi-square test or Fisher’s exact test for categorical variables, as appropriate. A two-sided *p* value < 0.05 was considered statistically significant. All statistical analyses were performed using R version 4.2.3.

### 2.4. Collinearity Assessment and Logistic Regression

Multicollinearity among retained modeling variables was examined using variance inflation factors (VIF). Logistic regression was then used to estimate univariable and multivariable associations with binary recurrence status. Continuous predictors were standardized where indicated by the forest plot, and model estimates were reported as odds ratios with 95% confidence intervals and two-sided *p* values.

The collinearity screen was applied before model interpretation because several composite indicators share clinical or laboratory components. A high degree of collinearity would make coefficient-level interpretation unstable and could obscure whether a composite index contributed information beyond its related variables. Variables with acceptable VIF profiles were therefore retained for subsequent modeling, with priority given to composite indicators when they summarized broader biological information. The multivariable logistic model was used as an interpretable baseline rather than as the only prediction model, allowing the subsequent survival-learning stage to address censoring and time-to-event structure.

### 2.5. Survival Model Development and Validation

RFS models were developed using a Mime-based machine-learning survival workflow similar to recent interpretable prognostic modeling strategies for PCa [24,25]. The cohort was randomly divided into a training set and an internal validation set at a ratio of 7:3 with stratification by BCR status, using caret::createDataPartition with stratification by recurrence status (seed = 5201314). After excluding patient identifiers, time-to-event variables and outcome labels, categorical variables were converted into dummy variables using model.matrix, and all candidate predictors were standardized according to the mean and standard deviation estimated from the training cohort.

Model development was performed using the Mime package in R. Candidate model architectures combined Cox-based feature-selection strategies, including bidirectional and backward stepwise Cox selection and penalized Cox selection, with survival machine-learning algorithms such as random survival forest and plsRcox. The ML.Dev.Prog.Sig function was used with candidate_genes, the Mime argument for input predictors, set to all transformed candidate predictors. The main parameters were as follows: unicox.filter.for.candi = FALSE, unicox_p_cutoff = 0.05, mode = “all”, nodesize = 5, seed = 5201314 and cores_for_parallel = 1. Model discrimination was summarized using the concordance index in the training and internal validation cohorts.

Time-dependent AUC values were calculated using cal_AUC_ml_res with mode = “all”, AUC_time = 1 or 2 years and auc_cal_method = “KM”. The leading model according to the Mime C-index results was selected for subsequent calibration, decision curve analysis, Kaplan–Meier stratification, and SHAP-based interpretation.

### 2.6. Calibration, Decision Curve Analysis, and Binary Classification Metrics

Calibration curves were used to compare predicted and observed 2-year recurrence risk. Decision curve analysis (DCA) was used to estimate net clinical benefit across threshold probabilities. For the five leading C-index models, binary performance metrics were calculated against the observed recurrence outcome at Youden-index thresholds and reported as sensitivity, specificity, and F1 score.

The Youden-index threshold was used as a data-driven operating point for binary recurrence classification assessments. Sensitivity, specificity, and F1 score were not treated as replacements for survival-model evaluation, because recurrence risk is inherently time dependent; instead, these metrics provided a familiar clinical summary of how each model behaved when reduced to a two-class decision using the observed outcome label. Calibration and DCA were evaluated for the top five C-index models in both the training and internal validation sets to determine whether high-ranking models retained reasonable absolute-risk behavior and potential net benefit beyond discrimination alone.

### 2.7. Model Interpretation and Clinical Cutoff Identification

Feature contributions were interpreted using survival SHAP analysis with the survex framework [26]. For continuous composite indicators, SHAP dependence plots were smoothed using locally weighted scatterplot smoothing. Clinical cutoffs were identified by linear interpolation at the point where the smoothed SHAP curve crossed the zero baseline, representing a transition between protective and risk-increasing model contributions. Patients were then stratified according to these cutoffs, and survival differences were evaluated using Kaplan–Meier curves, log-rank tests, and Cox regression.

### 2.8. Comparison of Performance Among Different Nested Models

We repeated the StepCox[both] + RSF workflow using ISUP Grade Groups 1–5 and pT2/pT3a/pT3b/pT4 as candidate predictors, retaining the original data split, other predictors and outcomes. Training-based standardization and indicator-level AIC selection were repeated, followed by RSF fitting to obtain the detailed pathology model. To assess the incremental value of PNI and LMR, we fitted two RSF models using the original training-validation split: a pathology-only model including ISUP Grade Group, pT stage, surgical margin, perineural invasion, lymphovascular invasion, and lymph-node status, and an otherwise identical model adding continuous PNI and LMR. Both used identical forest settings without further variable selection. The original and detailed pathology models were retained as benchmarks. Validation performance was assessed using C-index, 1–3-year AUC, Brier score and calibration; paired differences and 95% confidence intervals (CI) were estimated using 1000 validation-cohort bootstrap samples with fitted models held fixed.

### 2.9. GeneCards-Based Target Retrieval and Intersection Analysis

To connect the clinical index model with potential molecular targets, target retrieval was performed via the GeneCards database for three entities: postoperative BCR of PCa, LMR and PNI score [27]. For each entity, the GeneCards symbol, gene name, type, and relevance score were retained, and duplicate records were removed according to gene symbol. The resulting target lists were exported as Supplementary Tables S3–S5 to keep the target-discovery step transparent and auditable.

Overlapping targets were then identified by intersecting the BCR, LMR, and PNI score target lists. The overlap analysis was used to focus on genes that were simultaneously related to the disease outcome and both model-relevant composite indicators, rather than genes associated with only one component of the workflow. For each overlapping gene, the BCR relevance score, LMR relevance score, and PNI relevance score were preserved, and an average relevance score was calculated across the three sources. The final intersection table was provided as Supplementary Table S6. Genes were ranked by average relevance score, and the two top-ranked targets, TP53 and PTEN, were selected for downstream drug-sensitivity prediction.

This target-selection rule was intentionally conservative. GeneCards relevance scores are database-derived annotations rather than experimental measurements in the present cohort, so they were not used as evidence of causality. Instead, they were used to prioritize genes with simultaneous database support across recurrence, LMR, and PNI score. The resulting overlap was treated as a hypothesis-generating molecular layer, and the subsequent drug-sensitivity and docking analyses were interpreted as exploratory validation of candidate target-drug pairs rather than as direct clinical therapeutic evidence.

### 2.10. Drug Sensitivity Prediction and Molecular Docking

Potential drugs associated with TP53 and PTEN were screened by correlating gene expression with drug activity Z scores from the CellMiner resource [28]. For each gene, drugs approved by regulatory agencies or under clinical development were prioritized where possible, and the three strongest positive correlations were retained for visualization. The purpose of this step was not to infer therapeutic efficacy directly, but to nominate drug-target pairs whose pharmacological activity patterns were concordant with target expression across the CellMiner cell-line panel.

Molecular docking was performed for the six gene–drug pairs using AutoDock Vina (version 1.1.2) [29]. Receptor and ligand structures were prepared by removing non-essential molecules, adding hydrogens and assigning charges before docking, following established docking-practice recommendations [30,31]. Docking affinities were used as a structural plausibility filter rather than as proof of binding or biological activity. Binding poses were visualized in PyMOL (version 3.2.0), and two-dimensional interaction diagrams were generated in Discovery Studio to display the predicted local interaction patterns.

To complement the docking analysis, AlphaFold3-predicted PTEN and TP53 structures were used as additional receptor conformations [32]. The same six candidate ligands were prepared as described above and redocked using AutoDock Vina with the same main settings (exhaustiveness = 16 and num_modes = 9). The best-scoring poses were visualized in PyMOL.

## 3. Results

### 3.1. Clinicopathological and Composite Inflammatory Indicators Distinguish BCR Status

The analytical cohort comprised 320 PCa patients, including 195 patients in the non-recurrence group and 125 patients in the recurrence group. After a median follow-up of 38 months, the median time to recurrence was 6 months (interquartile range, 4–11 months) among patients who experienced BCR. For patients without BCR, the median observation time to censoring was 37 months (interquartile range, 25–55 months). Baseline age and BMI were similar between groups, whereas recurrence was associated with adverse pathological features, consistent with previous evidence linking lymphovascular invasion, perineural invasion, and positive surgical margin to BCR [33,34]. High D’Amico risk classification was more frequent in the recurrence group (70.40% vs. 38.46%), as were postoperative Gleason score ≥ 8 (57.60% vs. 22.57%), positive surgical margin (85.60% vs. 39.49%), perineural invasion (85.60% vs. 74.87%), lymphovascular invasion (43.20% vs. 15.90%), positive lymph node status (10.40% vs. 3.08%), and T3-T4 stage (52.00% vs. 29.23%) (all *p* < 0.05; Table 1). It is noteworthy that the high proportion of positive surgical margins in our study was largely driven by the high percentage of intermediate-risk and high-risk localized PCa patients with locally advanced disease.

Composite inflammatory or nutritional indicators also separated the two outcome groups, in line with prior work demonstrating the prognostic value of systemic inflammatory and nutritional indices in PCa. The recurrence group exhibited higher levels of LAR, PCD, LB-PSAD, PIV and PSA-PNI, whereas PNI score and LMR were lower than in the non-recurrence group (all *p* < 0.05; HPR did not differ significantly; Table 1). These findings supported the decision to retain composite markers in subsequent modeling rather than relying solely on their individual laboratory constituents.

These baseline patterns further indicated that the composite indicators were not merely duplicating a single pathological feature. LB-PSAD and PSA-PNI increased in the recurrence group, whereas PNI score and LMR decreased, indicating that recurrence was accompanied by both tumor-burden-related and host-condition-related signals. This distinction was important for the downstream modeling strategy. Variables such as postoperative Gleason score, surgical margin, lymphovascular invasion, and T stage captured local tumour aggressiveness, whereas PIV, PNI score, LMR, and LB-PSAD summarized immune, nutritional, metabolic, or PSA-density information. The coexistence of these patterns argued for a model that retained both clinicopathological variables and selected composite indicators, because removing either layer would have narrowed the biological and clinical information available to the model.

### 3.2. Collinearity Diagnostics and Logistic Regression Define an Interpretable Recurrence-Associated Feature Set

**Figure 1:**
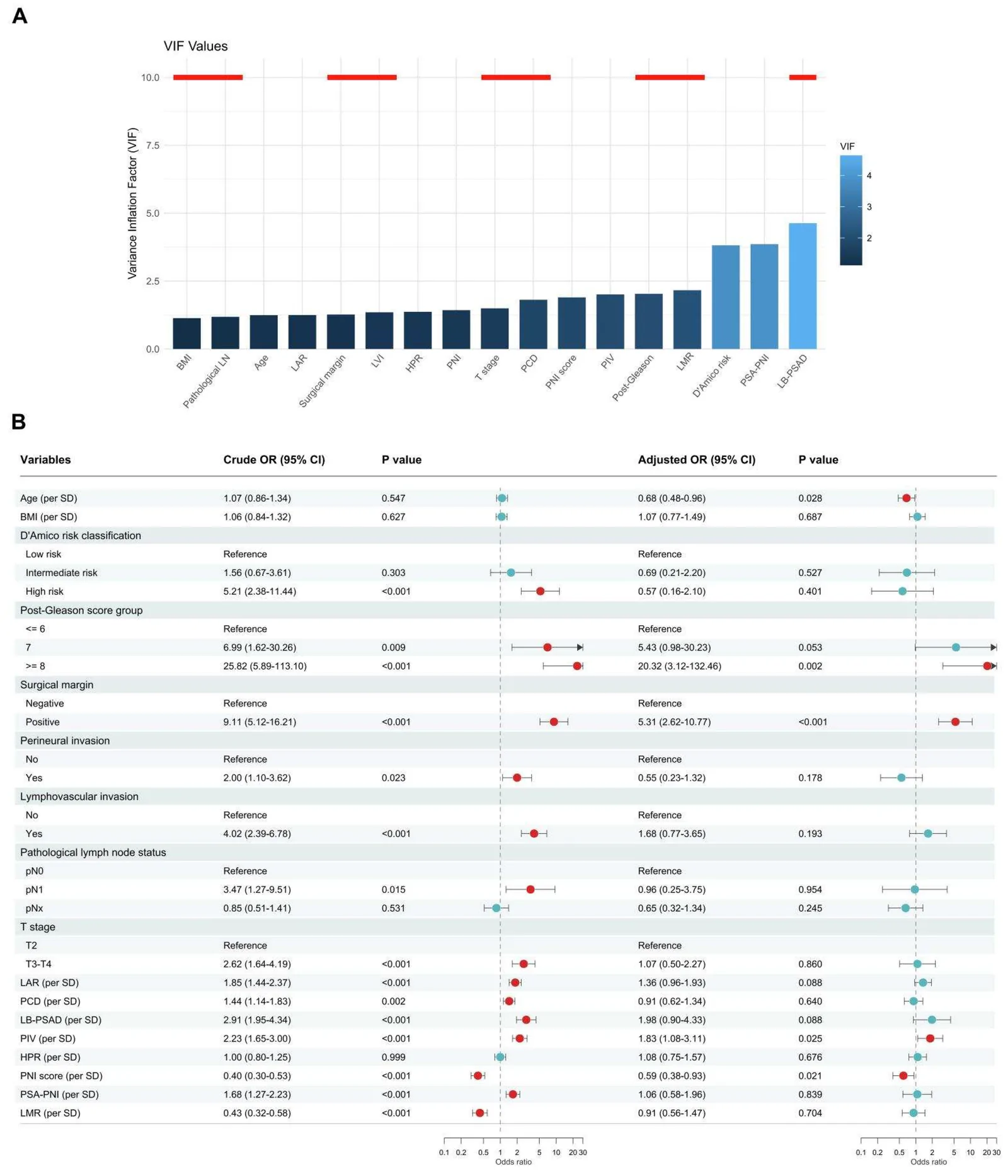
Collinearity diagnostics and logistic regression for recurrence-associated variables. (**A**) Variance inflation factor plot for the retained modeling variables. All variables remained below conventional high-collinearity thresholds. (**B**) Univariable and multivariable logistic regression forest plot for binary biochemical recurrence status. Red points denote statistically significant associations (*p* < 0.05), and blue points denote non-significant associations. OR, odds ratio; CI, confidence interval; PIV, pan-immune-inflammation value; PNI, prognostic nutritional index.

VIF analysis indicated that the retained variables did not show severe multicollinearity. All VIF estimates remained below conventional high-collinearity thresholds, with the largest values observed for LB-PSAD and related composite indices (Figure 1A). This supported joint evaluation of clinicopathological variables and composite biomarkers in the prediction model.

In logistic regression, several adverse pathological features and composite indicators were associated with recurrence in univariable analyses. In the multivariable model, age per standard deviation [adjusted odds ratio (OR), 0.68; 95% CI, 0.48–0.96; *p* = 0.028], postoperative Gleason score ≥ 8 (adjusted OR, 20.32; 95% CI, 3.12–132.46; *p* = 0.002), positive surgical margin (adjusted OR, 5.31; 95% CI, 2.62–10.77; *p* < 0.001), PIV per standard deviation (adjusted OR, 1.83; 95% CI, 1.08–3.11; *p* = 0.025) and PNI score per standard deviation (adjusted OR, 0.59; 95% CI, 0.38–0.93; *p* = 0.021) remained independently associated with BCR (Figure 1B).

The multivariable model therefore provided an interpretable bridge between conventional pathology and composite biomarkers. Postoperative Gleason score ≥8 and positive surgical margin represented high-risk tumor biology and incomplete local control, while PIV and PNI score represented systemic inflammatory-immune and nutritional status. Their simultaneous retention after adjustment suggested that recurrence risk was not captured by surgical pathology alone. At the same time, the model did not retain every significant univariable marker, which helped guard against overinterpreting correlated variables. In this sense, the logistic analysis functioned as a clinically readable companion to the subsequent machine-learning survival models: it identified a smaller set of independent recurrence-associated features while preserving the broader candidate set for time-to-event modeling.

### 3.3. Mime Survival Modeling Identifies StepCox Combined with Random Survival Forest as the Optimal Prognostic Framework

**Figure 2:**
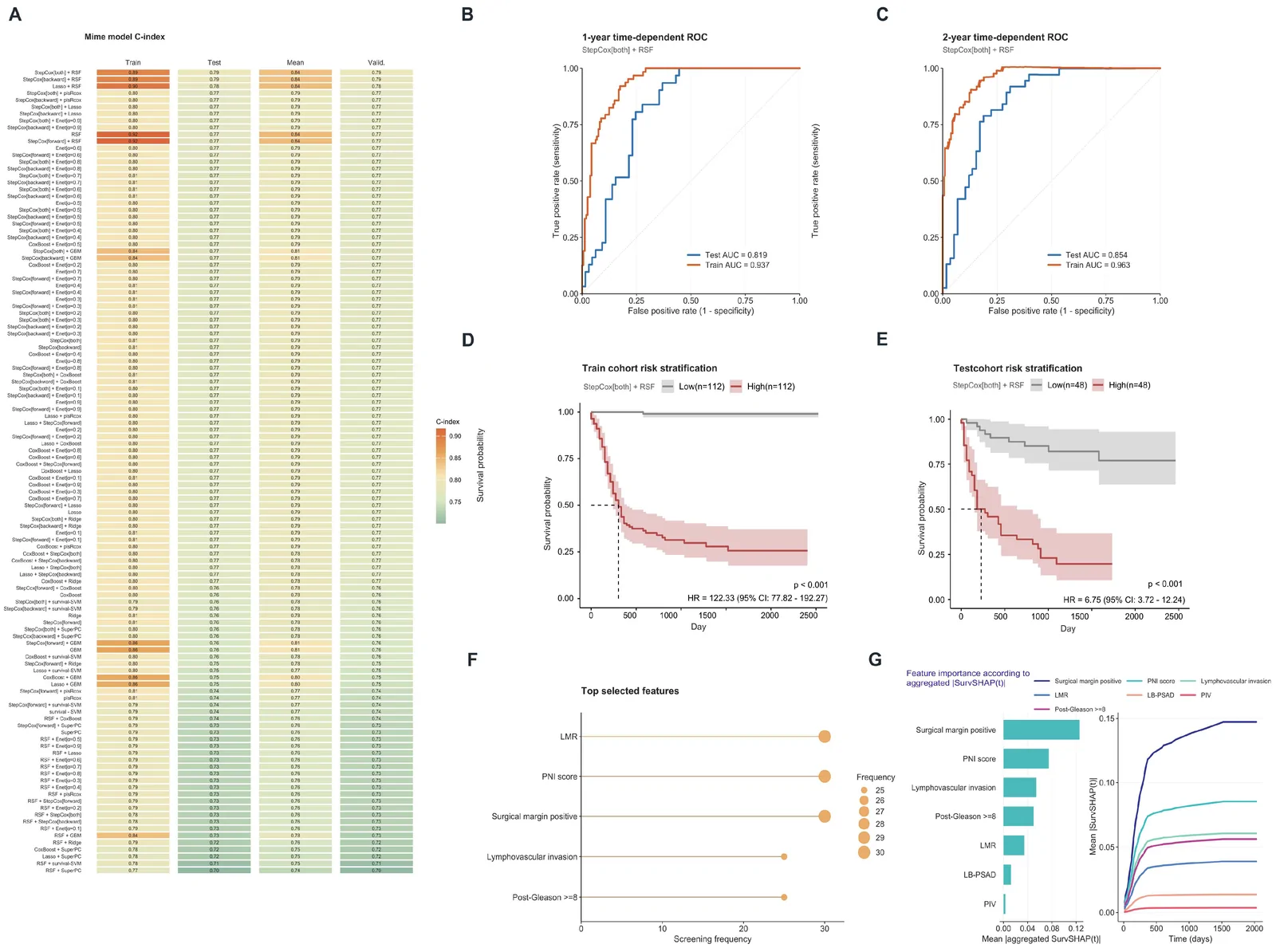
Mime survival modeling and interpretation of recurrence-free survival prediction. (**A**), C-index heatmap for survival model combinations evaluated in the Mime workflow. (**B**,**C**) Time-dependent ROC curves for the selected StepCox[both] + random survival forest model at 1 year and 2 years. (**D**,**E**) Kaplan–Meier curves comparing high- and low-risk groups in the training and test sets. (**F**) Frequency of core features selected by the leading model set. (**G**) SurvSHAP feature-importance visualization for the selected survival model. Abbreviations: PNI, prognostic nutritional index; LB-PSAD, lymphocyte-based prostate-specific antigen density; PIV, pan-immune-inflammation value; LMR, lymphocyte-to-monocyte ratio.

To evaluate RFS, a Mime survival-modeling workflow was used to compare Cox-based feature selection strategies combined with machine-learning survival learners. The leading architectures were StepCox[both] + random survival forest (RSF) and StepCox[backward] + RSF, each achieving a C-index of 0.788 in the internal validation cohort and a mean C-index of 0.840 across the training and internal validation cohorts (Figure 2A and Table 2). Lasso + RSF ranked immediately after these models, followed by StepCox combined with plsRcox.

The selected StepCox[both] + RSF model showed strong time-dependent discrimination. The 1-year area under the curve (AUC) was 0.937 in the internal validation set and 0.819 in the test set, and the 2-year AUC was 0.963 and 0.854, respectively (Figure 2B,C). Risk scores from the model separated patients into distinct recurrence-free survival strata in both the training cohort [hazard ratio (HR), 122.33; 95% CI, 77.82–192.27; *p* < 0.001] and the internal validation cohort (HR, 6.75; 95% CI, 3.72–12.24; *p* < 0.001; Figure 2D,E).

Across top-performing models, LMR, PNI score, and positive surgical margin showed the highest selection frequency, followed by lymphovascular invasion and postoperative Gleason score ≥ 8 (Figure 2F). SurvSHAP interpretation further highlighted positive surgical margin, PNI score, lymphovascular invasion, postoperative Gleason score ≥ 8, LMR, LB-PSAD, and PIV as major contributors to model risk over time (Figure 2G).

This convergence was important for the downstream biological analysis. The survival models repeatedly selected two composite indicators, LMR and PNI score, that also showed clinically meaningful SHAP-derived thresholds and strong survival separation. We therefore treated LMR and PNI score not only as statistical predictors but also as index-level anchors for target retrieval.

### 3.4. Calibration, Decision Curves and SHAP-Derived Thresholds Support Clinical Stratification

**Figure 3:**
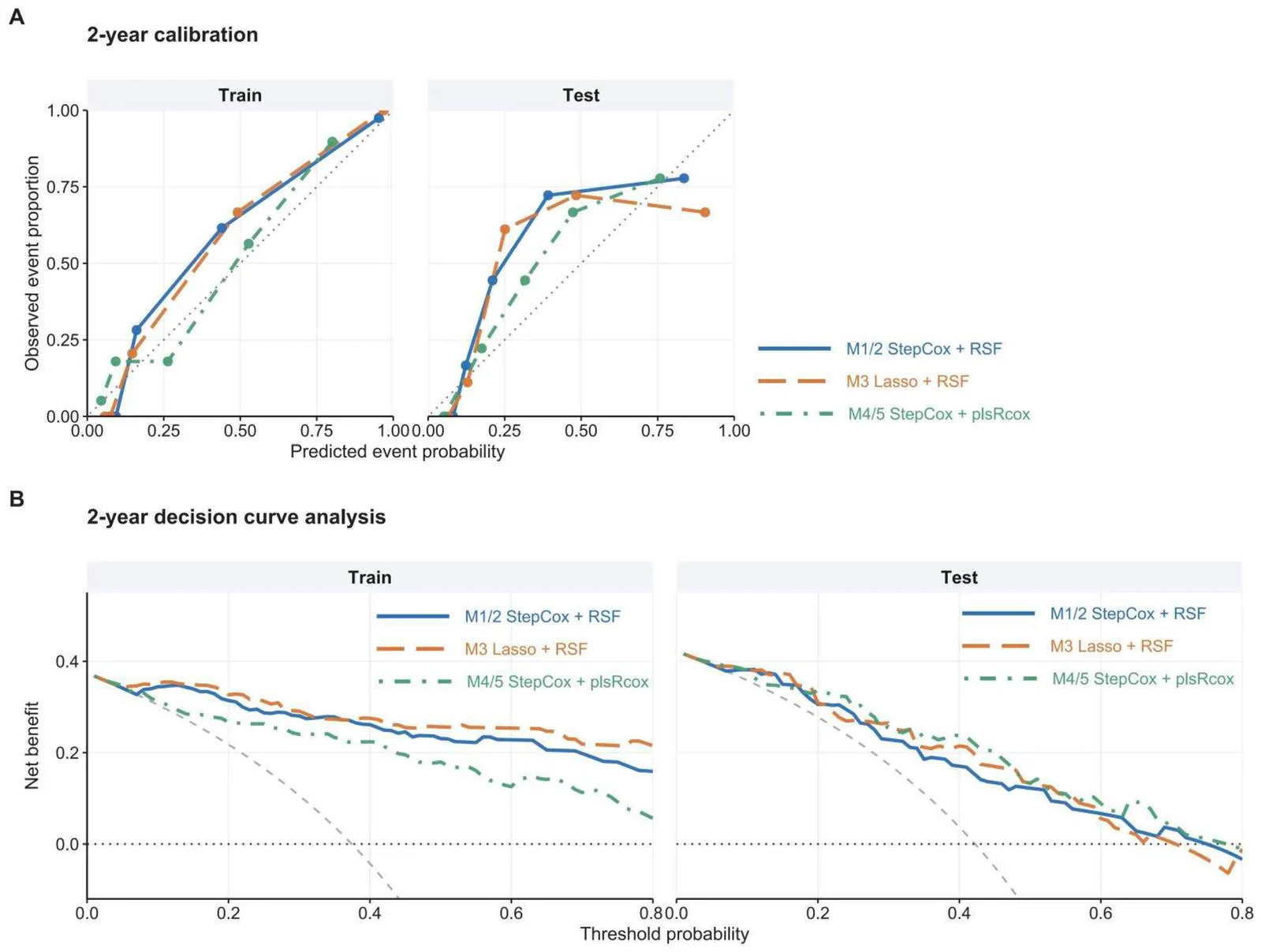
Calibration and decision curve analysis for the top five survival models. (**A**) Two-year calibration curves for StepCox[both] + RSF, StepCox[backward] + RSF, Lasso + RSF, StepCox[both] + plsRcox and StepCox[backward] + plsRcox in training and test sets. (**B**) Decision curve analysis for the same five models, showing net clinical benefit across threshold probabilities. M1/2 indicates the two StepCox + RSF models; M3 indicates Lasso + RSF; M4/5 indicates the two StepCox + plsRcox models.

For the five leading C-index models, 2-year calibration curves showed broadly acceptable agreement between predicted and observed risk in both the training and internal validation sets, and decision curve analysis suggested net clinical benefit across a clinically relevant range of threshold probabilities (Figure 3). Classification metrics calculated from the binary recurrence outcome at Youden-index cutoffs provided complementary summaries; for example, StepCox[both] + RSF achieved test-set sensitivity of 0.761 in the internal validation set, specificity of 0.880 and F1 score of 0.805, whereas Lasso + RSF showed higher sensitivity but lower specificity in the internal validation set (Table 2).

The side-by-side presentation of calibration, decision curves, and Youden-index metrics was intended to separate three different performance questions. Discrimination evaluated whether a model ranked patients correctly, calibration assessed whether the predicted absolute risks were numerically plausible, and decision curve analysis asked whether the model would provide net benefit if used to guide a threshold-based clinical decision. The leading models did not behave identically across these summaries. Lasso + RSF emphasized sensitivity in the internal validation set, while StepCox-based RSF models showed a more specific classification profile. This trade-off is clinically relevant: a screening-oriented tool may prefer sensitivity, whereas a model used to intensify surveillance or treatment discussions may require higher specificity to avoid unnecessary escalation.

**Figure 4:**
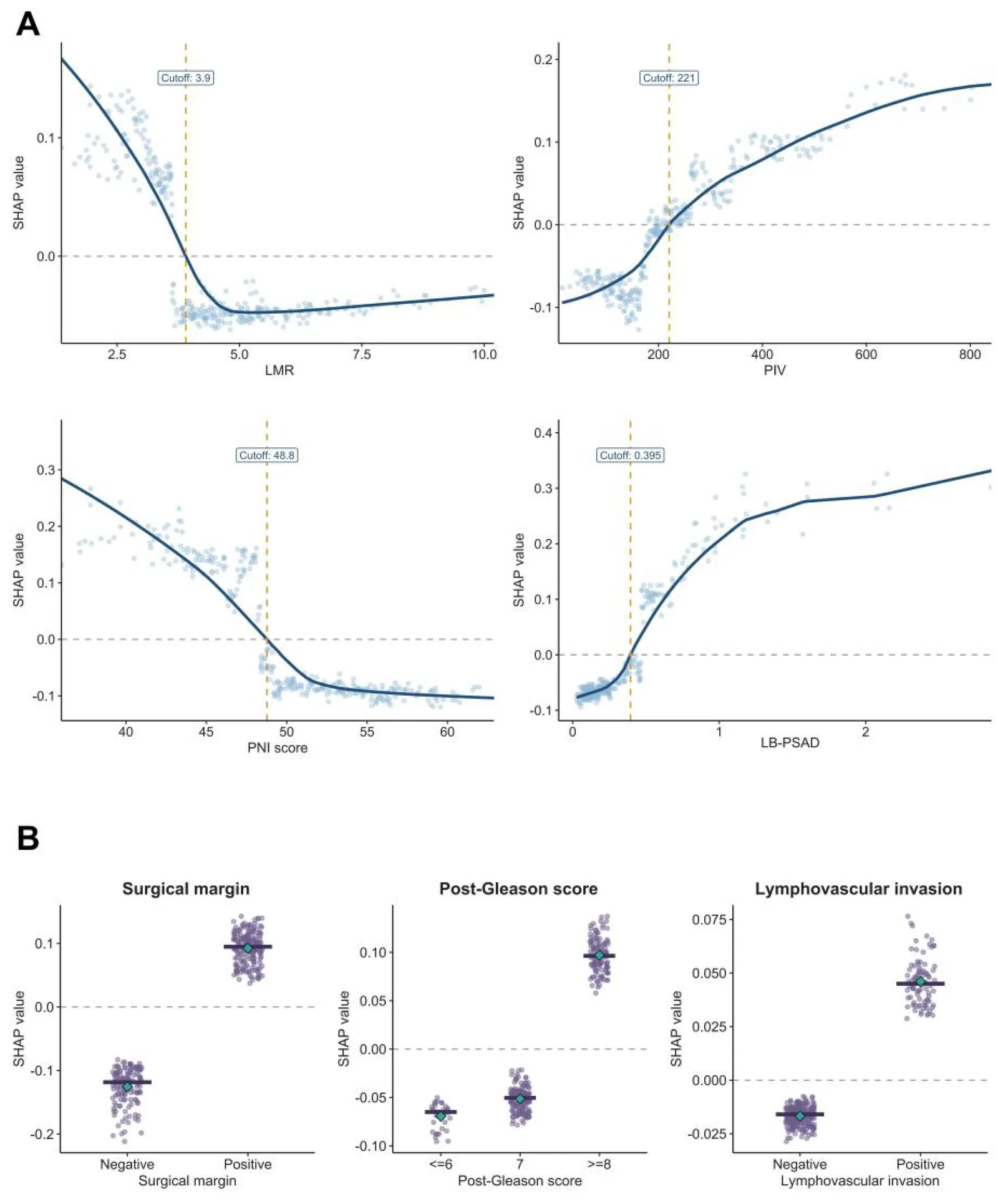

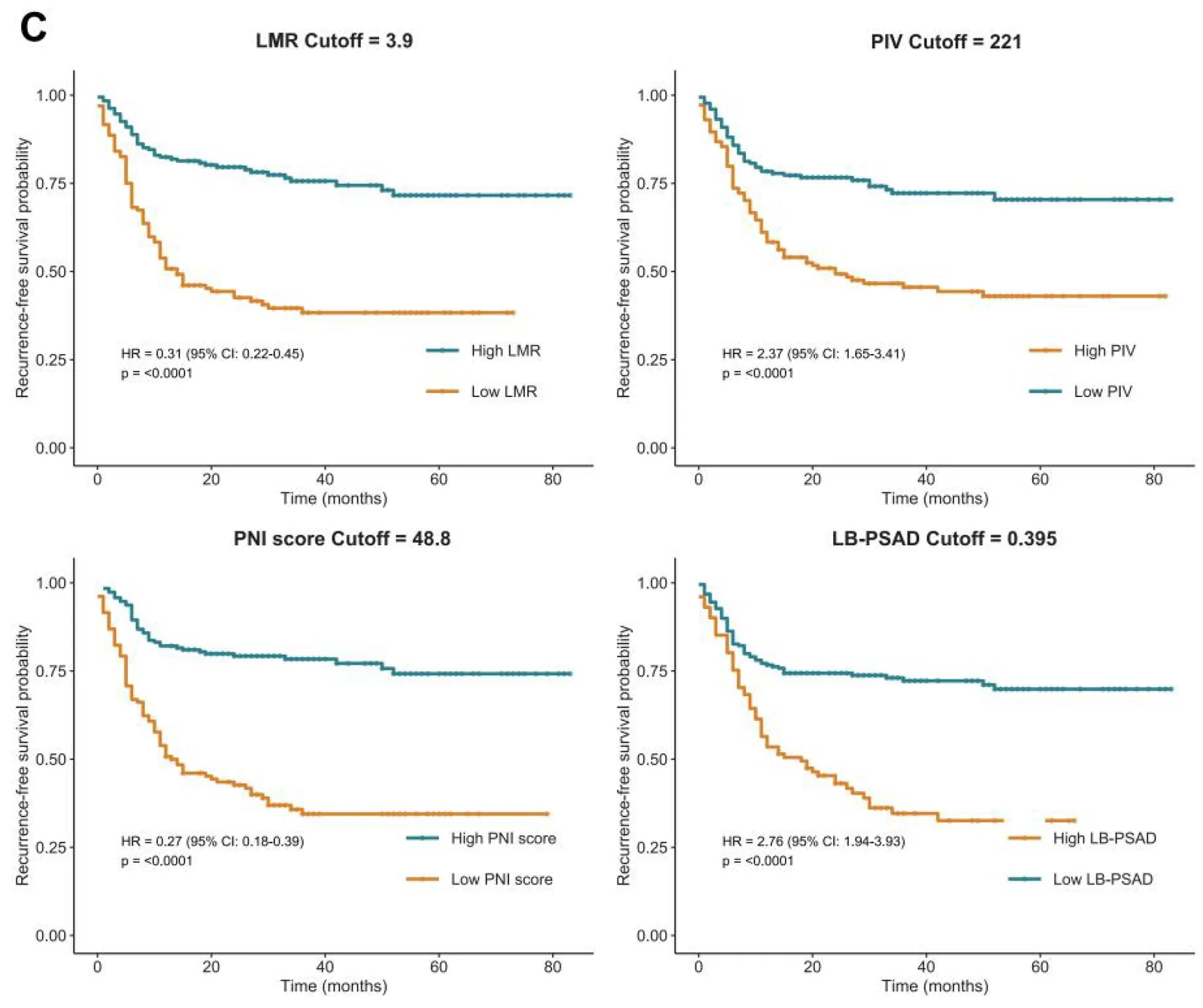
SHAP-derived cutoffs and clinical stratification of composite indicators. (**A**) SHAP dependence plots for LMR, PIV, PNI score, and LB-PSAD with LOWESS curves and zero-SHAP cutoffs. (**B**) SHAP dependence plots for categorical variables, including surgical margin, post-operative Gleason score, and lymphovascular invasion. (**C**), Kaplan–Meier curves comparing recurrence-free survival after stratification by the four composite-indicator cutoffs.

To convert continuous composite indicators into clinically readable strata, SHAP dependence curves were smoothed using locally weighted regression, and cutoffs were defined at the zero crossing of SHAP values. The derived cutoffs were 3.9 for LMR, 221 for PIV, 48.8 for PNI score, and 0.395 for LB-PSAD (Figure 4A). These thresholds produced consistent survival separation: higher LMR and PNI score were associated with better RFS, whereas higher PIV and LB-PSAD were associated with poorer RFS (all log-rank *p* < 0.0001; Figure 4C).

Categorical SHAP plots indicated that positive surgical margin, postoperative Gleason score ≥ 8, and lymphovascular invasion increased model-predicted recurrence risk (Figure 4B). Correlation analyses based on the four composite-indicator cutoffs further linked these strata to adverse clinicopathological patterns. Low LMR and low PNI score groups showed higher frequencies of high D’Amico risk, positive surgical margin and lymphovascular invasion; high PIV was associated with positive surgical margin and lymphovascular invasion, and high LB-PSAD captured a particularly adverse phenotype involving D’Amico risk, postoperative Gleason score, surgical margin, perineural invasion, lymphovascular invasion, lymph-node status, and T stage (Table 3 and Table 4, Supplementary Tables S1 and S2).

These cutoff analyses gave the model outputs a form that could be checked against routine clinical reasoning. A low LMR or low PNI score did not simply define a statistical subgroup; each threshold identified patients with worse recurrence-free survival and a higher prevalence of adverse pathological features. Conversely, high PIV and high LB-PSAD defined risk-enriched groups that were concordant with poorer survival. This agreement between SHAP-derived thresholds, Kaplan–Meier separation, and clinicopathological enrichment reduced the chance that the cutoffs were only mathematical artifacts of the fitted model.

### 3.5. Comparison of Performance Among Different Nested Models

In the validation cohort, the C-index increased from 0.740 for the pathology-only model to 0.786 after adding PNI and LMR (difference, 0.046; 95% CI, −0.004 to 0.094). The original and detailed pathology models had C-indices of 0.788 and 0.795, respectively. The paired C-index difference between the detailed pathology and original models was 0.008 (95% CI, −0.014 to 0.030). Adding PNI and LMR increased 1-, 2-, and 3-year AUCs from 0.809, 0.817, and 0.775 to 0.816, 0.850, and 0.843. Corresponding Brier scores changed from 0.165, 0.179, and 0.210 to 0.166, 0.166, and 0.182. Both nested models underpredicted overall 3-year recurrence risk, indicating persistent calibration limitations (Figure S1). These findings suggest possible incremental value, with substantial uncertainty.

### 3.6. PTEN- and TP53-Linked Drug Sensitivity and Docking Analyses Nominate Exploratory Candidate Interactions

To extend the prognostic analysis toward drug-response hypotheses, the two index signatures were linked to a target-overlap analysis. GeneCards retrieval yielded 2649 candidate targets for LMR, 6779 targets for PNI score, and 3827 targets for postoperative BCR of PCa (Supplementary Tables S3–S5). Intersecting the three target lists produced 1211 shared genes (Supplementary Table S6). These overlapping genes represent a conservative target space in which the molecular annotations of recurrence, LMR, and PNI score converge rather than three independent lists considered in isolation.

The 1211 overlapping genes were ranked by the average of their GeneCards relevance scores across BCR, LMR, and PNI score. TP53 showed the highest average relevance score (407.00), followed by PTEN (374.79), KRAS (347.47), BRCA1 (345.70), and BRCA2 (333.07). Because TP53 and PTEN occupied the top two positions and are biologically interpretable prostate cancer-related tumor suppressor genes, they were selected for downstream drug-sensitivity screening and docking validation. This ranking step provided a transparent bridge from clinical composite indicators to a small, testable target set.

**Figure 5:**
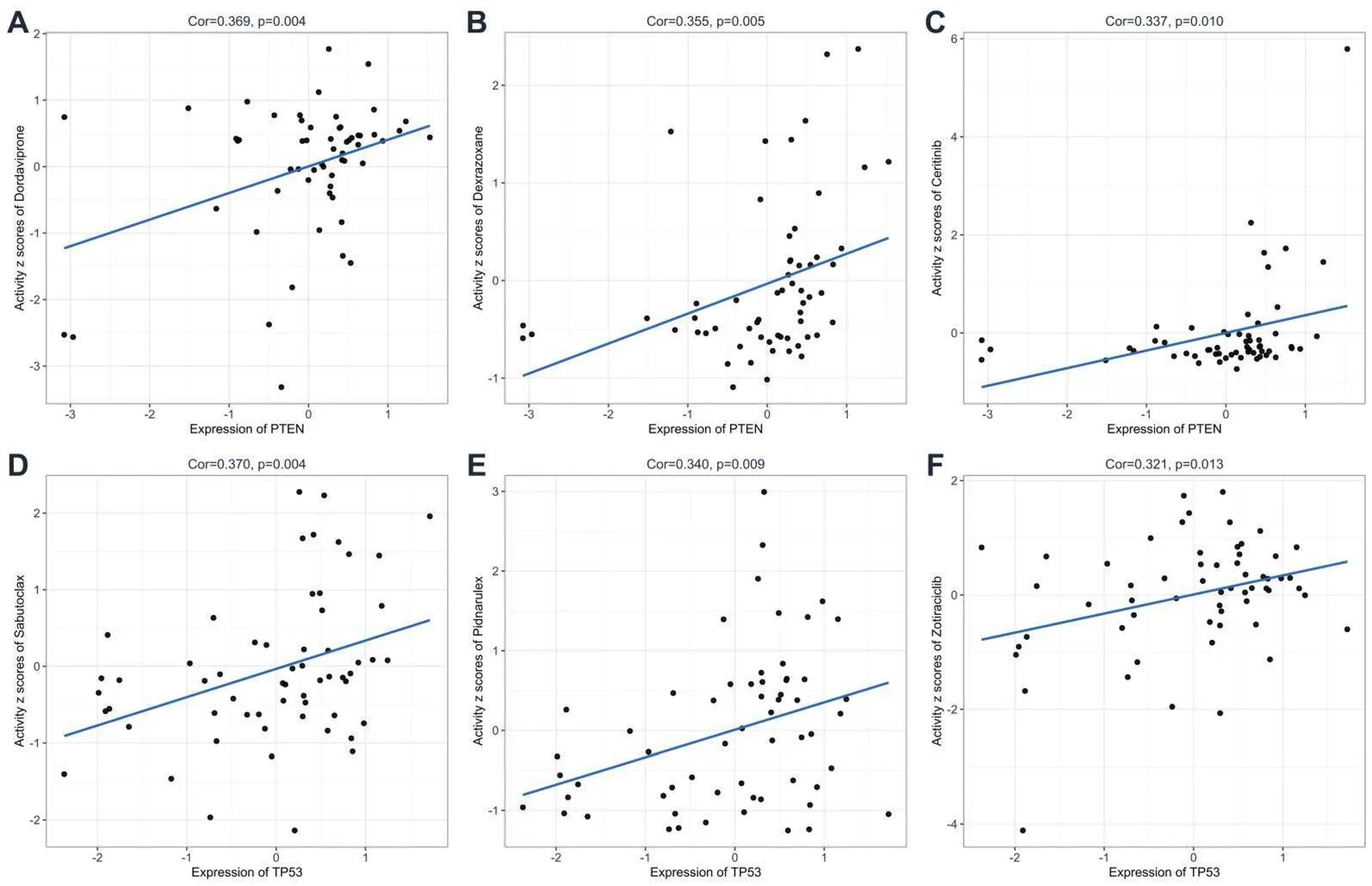
PTEN- and TP53-associated drug sensitivity prediction. (**A**–**C**) Scatter plots showing Pearson correlations between PTEN expression and drug activity z scores for the top three correlated drugs: Dordaviprone (**A**), Dexrazoxane (**B**), and Ceritinib (**C**). (**D**–**F**) Scatter plots showing Pearson correlations between TP53 expression and drug activity z scores for the top three correlated drugs: Sabutoclax (**D**), Pidnarulex (**E**), and Zotiraciclib (**F**).

CellMiner-based drug sensitivity analysis was then performed for PTEN and TP53. PTEN expression was positively correlated with the activity scores of dordaviprone (r = 0.369, *p* = 0.004), dexrazoxane (r = 0.355, *p* = 0.005), and ceritinib (r = 0.337, *p* = 0.010). TP53 expression was positively correlated with the drug sensitivity Z-scores of sabutoclax (r = 0.370, *p* = 0.004), pidnarulex (r = 0.340, *p* = 0.009), and zotiraciclib (r = 0.321, *p* = 0.013; Figure 5).

**Figure 6:**
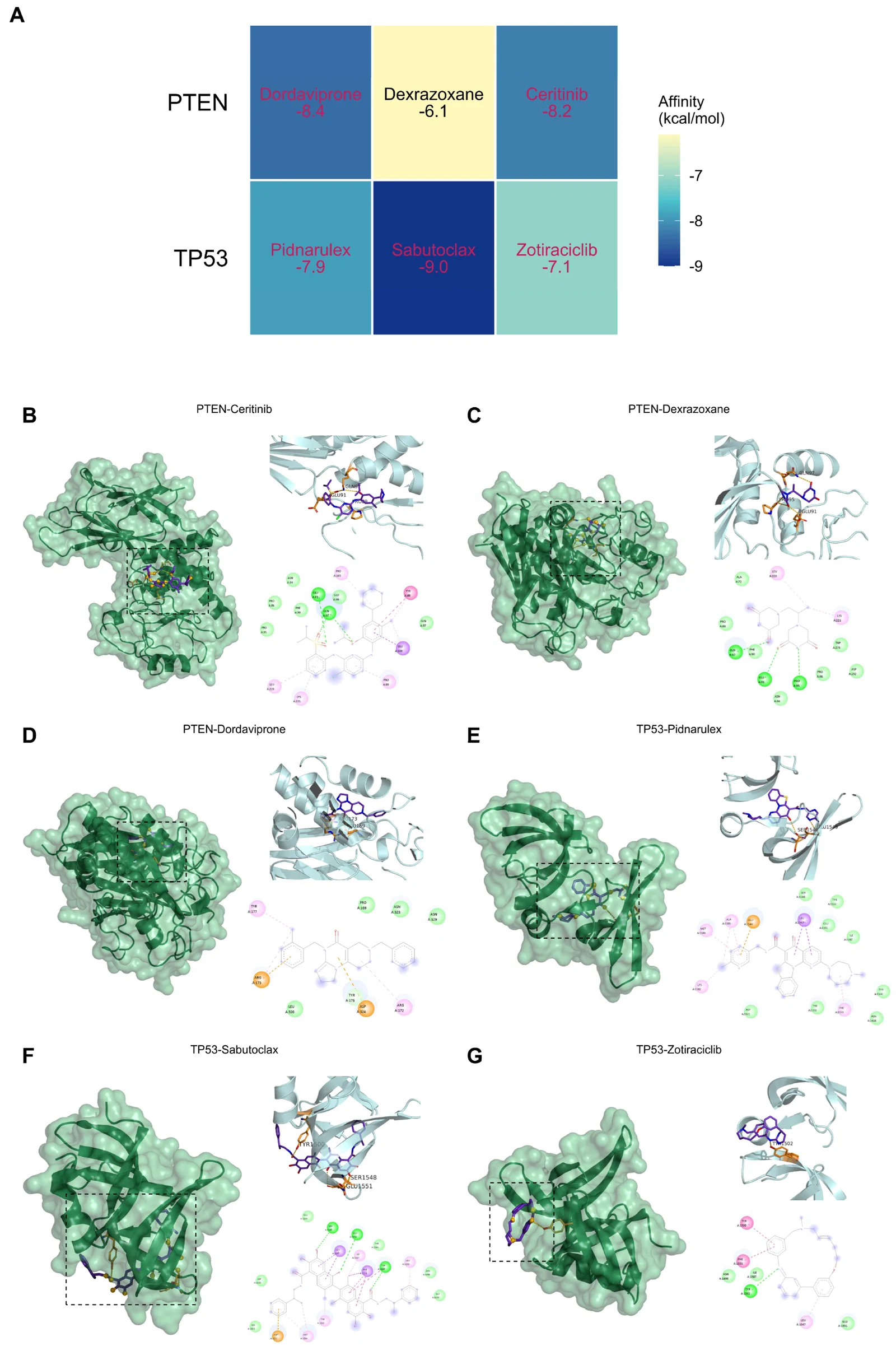
Molecular docking of PTEN- and TP53-associated candidate drugs. (**A**) Heatmap of AutoDock Vina binding affinities for six gene-drug docking pairs. (**B**–**G**) PyMOL-based three-dimensional docking views and two-dimensional ligand-interaction diagrams for PTEN-Dordaviprone, PTEN-Dexrazoxane, PTEN-Ceritinib, TP53-Pidnarulex, TP53-Sabutoclax, and TP53-Zotiraciclib.

Molecular docking was then used as an exploratory structural filter for these six gene-drug pairs. AutoDock Vina affinities ranged from −9.0 to −6.1 kcal/mol, with the strongest score observed for TP53-sabutoclax (−9.0 kcal/mol), followed by PTEN-dordaviprone (−8.4 kcal/mol), PTEN-ceritinib (−8.2 kcal/mol), TP53-pidnarulex (−7.9 kcal/mol), TP53-zotiraciclib (−7.1 kcal/mol) and PTEN-dexrazoxane (−6.1 kcal/mol; Figure 6A). Three-dimensional and two-dimensional interaction diagrams visualized the predicted binding poses and polar contacts (Figure 6B–G). These drug analyses should be interpreted as hypothesis-generating and require experimental validation.

As a complementary analysis, docking was repeated using the AlphaFold3-predicted PTEN and TP53 receptor structures. The AlphaFold3 receptor-based Vina affinities ranged from −10.4 to −6.6 kcal/mol, with the strongest score observed for TP53-sabutoclax (−10.4 kcal/mol), followed by PTEN-dordaviprone and TP53-pidnarulex (−8.4 kcal/mol each), PTEN-ceritinib (−8.0 kcal/mol), TP53-zotiraciclib (−7.9 kcal/mol) and PTEN-dexrazoxane (−6.6 kcal/mol; Figure S2A). PyMOL visualization showed the corresponding global binding poses and local polar-contact patterns for the six AlphaFold3 receptor-based docking pairs (Figure S2B–G), supporting the structural plausibility of the candidate interactions.

## 4. Discussion

Radical prostatectomy serves as the gold-standard treatment for localized PCa, which can prolong life expectancy and improve prognosis [35]. However, BCR occurs in approximately 30–40% of patients within 10–15 years after surgery [3]. Early identification of patients at high risk of recurrence, followed by individualized surveillance and intervention, is critical to improving long-term outcomes. Currently, most conventional prognostic stratification tools in clinical practice are based on tumor pathological features, with inadequate incorporation of systemic immune and nutritional status, and thus fail to capture the full biological spectrum underlying tumor progression. In this study, we retrospectively included 320 patients with PCa who underwent LRP, integrated a panel of composite systemic inflammatory and nutritional indices, and constructed a prognostic model for BCR using the Mime machine learning modeling pipeline. We comprehensively validated the model across three core performance dimensions: discrimination, calibration, and net clinical benefit. Furthermore, we derived clinically interpretable cutoffs for core indices via SHAP-based model interpretation, and explored potential molecular targets and candidate therapeutic agents anchored to the key phenotypic markers. Taken together, our findings provide a novel prognostic tool with both robust predictive performance and clinical utility for postoperative risk stratification in PCa, and delineate promising directions for subsequent translational research.

Systemic inflammation and nutritional status are key factors influencing tumorigenesis and cancer progression, and accumulating evidence has firmly established their close association with clinical outcomes across multiple malignancies [36,37,38]. In our cohort, patients with BCR not only exhibited more aggressive high-risk pathological features, including higher Gleason score, greater rates of positive surgical margins and lymphovascular invasion, but also showed altered systemic inflammatory and nutritional profiles. Specifically, these patients showed decreased LMR and PNI score, alongside elevated PIV and PSA density-related indices, suggesting that tumor progression is accompanied by remodeling of the host systemic microenvironment. After multivariable adjustment, elevated PIV and decreased PNI score remained independent risk factors for BCR. Notably, LMR and PNI score were consistently selected as core predictive features across all high-performing models, which further supports the robustness of their prognostic value. Notably, multivariable logistic regression identified higher age (per standard deviation) as an independent protective factor against BCR (adjusted OR = 0.68, *p* = 0.028). The inverse association between age and BCR likely stems from detection bias—a form of selection bias—wherein stringent preoperative screening in the elderly may exclude high-risk cases, whereas younger patients may present with more aggressive phenotypes.

PNI, calculated from serum albumin and total lymphocyte counts, reflects the host’s nutritional reserve and immune competence. Two meta-analyses have confirmed that a low baseline PNI score acts as an independent prognostic risk factor in men with PCa. Patients with decreased PNI tend to have shorter overall survival (OS) and progression-free survival, which lends robust evidence to support the clinical use of PNI score [10,11]. Among elderly patients with PCa, PNI score outperforms other nutritional scoring systems, including the nutritional risk index and CONUT score, for prognostic stratification, and exhibits better predictive efficacy than conventional AJCC staging [39]. For patients with metastatic hormone-sensitive PCa, PNI can effectively stratify clinical prognosis. Patients with a PNI score above 49.98 achieved markedly longer median OS. In addition, PNI score may help clinicians choose personalized endocrine treatment strategies [40]. Moreover, studies indicate that the PNI has predictive efficacy in bone metastasis and multiple bone metastases of PCa, with an AUC of 0.634 and a cutoff value of 44.925 [41]. Therefore, the PNI score demonstrates stable predictive capability across different stages of PCa. From a mechanistic perspective, a low PNI score reflects concurrent malnutrition and chronic systemic inflammation, which facilitates tumor proliferation, bone metastatic progression, and treatment resistance by altering the antitumor immune microenvironment, partially explaining why a reduced PNI score correlates with unfavorable oncological outcomes across localized and advanced PCa [42]. LMR serves as a proxy for the balance between anti-tumor immunity and pro-tumor inflammation. Mechanistically, lymphocytes serve as the primary effector cells mediating cellular antitumor immunity. Peripheral blood monocytes, conversely, migrate into the tumor microenvironment and differentiate into tumor-associated macrophages. Through secretion of proinflammatory cytokines and promotion of pathological angiogenesis, these cells drive tumor progression and facilitate immune evasion [43,44]. A meta-analysis demonstrated that the LMR constitutes an independent prognostic factor for long-term outcomes in patients with PCa. Compared with patients with a low pretreatment LMR, those with a higher pretreatment LMR exhibit superior prognosis [12]. Olcucu et al. reported an association between a lower LMR (<3.75) and shorter BCR-free survival in PCa patients who underwent robot-assisted radical prostatectomy [45]. These findings are consistent with our results: the recurrence group had lower PNI scores and LMR, and a lower PNI score was an independent risk factor for poor RFS.

Most conventional prognostic models for PCa are developed using Cox regression and visualized as nomograms. While they offer favorable interpretability, these models cannot fully capture complex nonlinear interactions among covariates, resulting in an inherent limit on predictive accuracy [46]. Over recent years, machine learning-based approaches have been increasingly applied to cancer prognostic modeling and have demonstrated enhanced predictive accuracy across the diagnosis, risk stratification, and clinical management of PCa [47]. Using the Mime pipeline, we systematically compared multiple combinations of feature selection strategies and survival learning algorithms, and identified the StepCox[both] combined with RSF as the optimal model. This model achieved a C-index of 0.788 in the validation cohort, with favorable performance in time-dependent AUC, calibration, and net clinical benefit. We next applied SurvSHAP analysis to quantify the time-dependent contribution of each covariate to risk prediction, mitigating the key interpretability limitation of machine learning models. Using the zero-baseline intersections of smoothed SHAP curves, we derived exploratory, model-derived cutoffs for four core indices (LMR, PIV, PNI score, and LB-PSAD). Unlike cutoffs defined solely by data distribution, these thresholds consistently achieved robust survival stratification and showed clear clinicopathological correlations, greatly reducing the risk of spurious findings driven by model overfitting. Our prognostic model may carry potential clinical implications. Hypothetically, it could help identify patients at higher risk of BCR who may warrant consideration for adjuvant radiotherapy or intensified postoperative PSA monitoring. Meanwhile, unlike the Decipher genomic classifier requiring tumor tissue for RNA profiling [48], our model relies on routine preoperative laboratory and pathological parameters without additional molecular testing, offering potential cost advantages and wider accessibility. It is not intended to replace genomic assays but can serve as a preliminary risk-screening tool, with high-risk patients further considered for complementary genomic testing if clinically indicated. Nevertheless, these potential applications remain exploratory. External validation is required before this model can be reliably used to guide clinical decisions.

Linking molecular targets and therapeutic candidates to clinical phenotypes represents a key step in translating prognostic biomarkers from bench to bedside. In this study, we leveraged the two most robust composite indices selected by our model—LMR and PNI—and integrated them with the BCR phenotype to perform target overlap analysis. Among 1211 shared genes, we identified TP53 and PTEN as the two tumor suppressor genes with the highest association scores. TP53 and PTEN are among the most frequently mutated and well-known tumor suppressor genes in PCa, and their loss of function is directly associated with genomic instability and increased tumor aggressiveness [49,50]. Mechanistically, these genes serve as crucial bridges linking systemic inflammatory-nutritional status to BCR. PTEN loss activates the PI3K/AKT signaling pathway, promoting the secretion of pro-inflammatory cytokines and recruiting immunosuppressive cells (e.g., tumor-associated macrophages), which directly remodels the tumor microenvironment to alter circulating leukocyte ratios (thereby affecting LMR and PNI) [51]. Similarly, TP53 mutations not only drive a proinflammatory tumor phenotype and dysregulate the tumor immune microenvironment, but also disrupt metabolic homeostasis and blunt cellular responses to nutritional stress, thereby exacerbating systemic nutritional impairment [52,53]. Consequently, the molecular profiles of TP53 and PTEN effectively translate the systemic milieu captured by our composite indices into the biological drivers of recurrence. Based on the drug sensitivity analysis of TP53 and PTEN expression, six candidate drugs were identified, and molecular docking results demonstrated favorable binding affinity for all of them. As a pan-BCL-2 family inhibitor, sabutoclax (BI-97C1) has exhibited potent anti-tumor effects by triggering mitochondrial apoptosis in preclinical prostate cancer models [54]. Pidnarulex (CX-5461), originally characterized as a selective RNA polymerase I inhibitor, exerts dual anti-tumor activity via G-quadruplex stabilization and topoisomerase II poisoning. This compound triggers extensive DNA damage and genomic instability, conferring synthetic lethality against tumors with defective DNA damage repair, including those harboring TP53 mutations or PTEN loss [55,56]. It is important to emphasize that these results are based on public databases and bioinformatics predictions, and they are hypothesis-generating in nature. Further in vitro and in vivo experiments are still needed to validate their actual efficacy and underlying mechanisms.

This study has several limitations. First, it is a single-center retrospective design, which may introduce selection bias. Second, the sample size was relatively small, and large-scale, prospective, multicenter studies are needed to further validate the performance and generalizability of our model across diverse regions and populations in the future. Third, both target selection and drug predictions were based on public databases and computational simulations, and were not validated in cellular or animal experiments.

## 5. Conclusions

We developed and validated an interpretable machine learning model incorporating systemic inflammatory and nutritional indices to predict BCR after RP. SHAP-derived cutoff values may facilitate individualized risk stratification for postoperative surveillance. Furthermore, integrated bioinformatic analyses identified TP53 and PTEN as pivotal links connecting these indices to disease recurrence, and molecular docking revealed sabutoclax and dordaviprone as potential therapeutic candidates. Prospective multicenter validation and experimental studies are warranted to corroborate these findings.

## Figures and Tables

**Table 1:** Comparison of model variables between non-recurrence and recurrence groups.

Characteristics	Non-Recurrence Group (*n* = 195)	Recurrence Group (*n* = 125)	t/Z/Chi-Square	*p* Value
Age	68.71 ± 6.71	69.17 ± 6.64	0.603	0.547
BMI [M(Q1, Q3)], kg/m^2^	25.16(23.29, 27.00)	25.51(23.81, 27.70)	1.164	0.244
D’Amico risk classification (*n*, %)			31.900	<0.001 *
Low risk	40(20.51)	9(7.20)		
Intermediate risk	80(41.03)	28(22.40)		
High risk	75(38.46)	88(70.40)		
Postoperative ISUP Grade Group (*n*, %)			52.357	<0.001 *
GG1 (Gleason ≤ 6)	32(16.41)	2(1.60)		
GG2 (Gleason 3 + 4)	68(34.87)	23(18.40)		
GG3 (Gleason 4 + 3)	51(26.15)	28(22.40)		
GG4 (Gleason 8)	20(10.26)	21(16.80)		
GG5 (Gleason 9–10)	24(12.31)	51(40.80)		
Surgical margin (*n*, %)			66.280	<0.001 *
Negative	118(60.51)	18(14.40)		
Positive	77(39.49)	107(85.60)		
Perineural invasion (*n*, %)			5.296	0.021 *
No	49(25.13)	18(14.40)		
Yes	146(74.87)	107(85.60)		
Lymphovascular invasion (*n*, %)			29.108	<0.001 *
No	164(84.10)	71(56.80)		
Yes	31(15.90)	54(43.20)		
Pathological lymph node status (*n*, %)			7.700	0.021 *
pN0	125(64.10)	78(62.40)		
pN1	6(3.08)	13(10.40)		
pNx	64(32.82)	34(27.20)		
Pathological T stage (*n*, %)			20.466	<0.001 *
pT2	138(70.77)	60(48.00)		
pT3a	25(12.82)	18(14.40)		
pT3b	25(12.82)	38(30.40)		
pT4	7(3.59)	9(7.20)		
LAR [M(Q1, Q3)]	3.97(3.59, 4.48)	4.39(3.97, 5.14)	4.937	<0.001 *
PCD [M(Q1, Q3)]	0.15(0.06, 0.25)	0.21(0.10, 0.30)	3.201	0.001 *
LB-PSAD [M(Q1, Q3)]	0.18(0.11, 0.32)	0.41(0.18, 0.76)	6.253	<0.001 *
PIV [M(Q1, Q3)]	176.30(127.19, 253.18)	263.92(187.03, 409.49)	5.601	<0.001 *
HPR [M(Q1, Q3)]	0.65(0.54, 0.75)	0.64(0.54, 0.74)	−0.270	0.787
PNI score [M(Q1, Q3)]	52.20(48.85, 55.52)	47.25(43.85, 51.25)	−6.985	<0.001 *
PSA-PNI [M(Q1, Q3)]	619.30(415.29, 866.91)	755.60(510.74, 1487.74)	3.379	<0.001 *
LMR [M(Q1, Q3)]	4.60(3.84, 5.74)	3.49(2.73, 4.42)	−6.765	<0.001 *

* p < 0.05 is considered statistically significant. Age is presented as mean +/− SD; other continuous variables are presented as median (Q1, Q3); categorical variables are presented as *n* (%). Abbreviations: BMI, body mass index; ISUP, International Society of Urological Pathology; GG, Grade Group; pT, pathological T stage; PNI, prognostic nutritional index; LAR, LDH-to-albumin ratio; PCD, positive core density; LB-PSAD, lymphocyte-based prostate-specific antigen density; PIV, pan-immune-inflammation value; HPR, hemoglobin-to-platelet ratio; LMR, lymphocyte-to-monocyte ratio.

**Table 2:** Comparison results of discrimination ability indicators for 5 models.

Model	Cohort	Validation Concordance Index	Youden Threshold	Sensitivity	Specificity	F1 Score
1. StepCox[both] + RSF	Train	0.788	6.533	0.937	0.855	0.851
1. StepCox[both] + RSF	Test	0.788	8.606	0.761	0.880	0.805
2. StepCox[backward] + RSF	Train	0.788	6.533	0.937	0.855	0.851
2. StepCox[backward] + RSF	Test	0.788	8.606	0.761	0.880	0.805
3. Lasso + RSF	Train	0.780	7.372	0.962	0.890	0.889
3. Lasso + RSF	Test	0.780	6.256	0.935	0.700	0.827
4. StepCox[both] + plsRcox	Train	0.773	0.682	0.772	0.862	0.762
4. StepCox[both] + plsRcox	Test	0.773	0.685	0.652	0.920	0.750
5. StepCox[backward] + plsRcox	Train	0.773	0.682	0.772	0.862	0.762
5. StepCox[backward] + plsRcox	Test	0.773	0.685	0.652	0.920	0.750

Abbreviations: F1 score, harmonic mean of precision and recall; LASSO, least absolute shrinkage and selection operator; plsRcox, partial least squares regression for Cox models; RSF, random survival forest; StepCox, stepwise Cox regression; Youden threshold, optimal cut-off value determined by the Youden index.

**Table 3:** Correlation analysis of patients with LMR < 3.90 and LMR ≥ 3.90 with clinicopathological features.

Characteristics	LMR < 3.90 (*n* = 132)	LMR ≥ 3.90 (*n* = 188)	t/Z/Chi-Square	*p* Value
Age	70.42 ± 6.37	67.81 ± 6.68	3.525	<0.001 *
BMI [M(Q1, Q3)], kg/m^2^	25.39(23.74, 27.26)	25.10(23.18, 27.20)	0.582	0.561
D’Amico risk classification (*n*, %)			12.881	0.002 *
Low risk	16(12.12)	33(17.55)		
Intermediate risk	33(25.00)	75(39.89)		
High risk	83(62.88)	80(42.55)		
Postoperative ISUP Grade Group (*n*, %)			13.349	0.010 *
GG1 (Gleason ≤ 6)	6(4.55)	28(14.89)		
GG2 (Gleason 3 + 4)	34(25.76)	57(30.32)		
GG3 (Gleason 4 + 3)	33(25.00)	46(24.47)		
GG4 (Gleason 8)	19(14.39)	22(11.70)		
GG5 (Gleason 9–10)	40(30.30)	35(18.62)		
Surgical margin (*n*, %)			28.157	<0.001 *
Negative	33(25.00)	103(54.79)		
Positive	99(75.00)	85(45.21)		
Perineural invasion (*n*, %)			1.675	0.196
No	23(17.42)	44(23.40)		
Yes	109(82.58)	144(76.60)		
Lymphovascular invasion (*n*, %)			9.420	0.002 *
No	85(64.39)	150(79.79)		
Yes	47(35.61)	38(20.21)		
Pathological lymph node status (*n*, %)			0.550	0.759
pN0	81(61.36)	122(64.89)		
pN1	9(6.82)	10(5.32)		
pNx	42(31.82)	56(29.79)		
Pathological T stage (*n*, %)			12.523	0.006 *
pT2	71(53.79)	127(67.55)		
pT3a	15(11.36)	28(14.89)		
pT3b	37(28.03)	26(13.83)		
pT4	9(6.82)	7(3.72)		

* means the *p*-value < 0.05 is considered statistically significant; BMI, body mass index; LMR, lymphocyte-to-monocyte ratio; ISUP, International Society of Urological Pathology; GG, Grade Group; pT, pathological T stage.

**Table 4:** Correlation analysis of patients with PNI score < 48.8 and PNI score ≥ 48.8 with clinicopathological features.

Characteristics	PNI Score < 48.8 (*n* = 130)	PNI Score ≥ 48.8 (*n* = 190)	t/Z/Chi-Square	*p* Value
Age	70.52 ± 6.69	67.77 ± 6.44	3.672	<0.001 *
BMI [M(Q1, Q3)], kg/m^2^	25.13(23.75, 27.04)	25.31(23.39, 27.55)	0.501	0.617
D’Amico risk classification (*n*, %)			12.660	0.002 *
Low risk	12(9.23)	37(19.47)		
Intermediate risk	37(28.46)	71(37.37)		
High risk	81(62.31)	82(43.16)		
Postoperative ISUP Grade Group (*n*, %)			10.174	0.038 *
GG1 (Gleason ≤6)	10(7.69)	24(12.63)		
GG2 (Gleason 3 + 4)	29(22.31)	62(32.63)		
GG3 (Gleason 4 + 3)	35(26.92)	44(23.16)		
GG4 (Gleason 8)	16(12.31)	25(13.16)		
GG5 (Gleason 9–10)	40(30.77)	35(18.42)		
Surgical margin (*n*, %)			36.530	<0.001 *
Negative	29(22.31)	107(56.32)		
Positive	101(77.69)	83(43.68)		
Perineural invasion (*n*, %)			0.116	0.733
No	26(20.00)	41(21.58)		
Yes	104(80.00)	149(78.42)		
Lymphovascular invasion (*n*, %)			8.736	0.003 *
No	84(64.62)	151(79.47)		
Yes	46(35.38)	39(20.53)		
Pathological lymph node status (*n*, %)			4.409	0.110
pN0	78(60.00)	125(65.79)		
pN1	12(9.23)	7(3.68)		
pNx	40(30.77)	58(30.53)		
Pathological T stage (*n*, %)			4.359	0.225
pT2	74(56.92)	124(65.26)		
pT3a	16(12.31)	27(14.21)		
pT3b	32(24.62)	31(16.32)		
pT4	8(6.15)	8(4.21)		

* means the *p*-value < 0.05 is considered statistically significant; BMI, body mass index; PNI, prognostic nutritional index; ISUP, International Society of Urological Pathology; GG, Grade Group; pT, pathological T stage.

## Data Availability

The original contributions presented in this study are included in the Supplementary Materials. Further inquiries can be directed to the corresponding author.

## Supplementary Materials

The following supporting information can be downloaded at: https://www.mdpi.com/article/10.3390/curroncol33100608/s1, Table S1: Correlation analysis of patients with PIV < 221 and PIV ≥ 221 with clinicopathological features.; Table S2: Correlation analysis of patients with LB-PSAD < 0.395 and LB-PSAD ≥ 0.395 with clinicopathological features. Table S3: lists LMR-associated GeneCards targets; Table S4: lists PNI score-associated targets; Table S5: lists postoperative biochemical recurrence-related GeneCards targets; Table S6: reports the intersected target set across BCR, LMR and PNI score, including separate relevance scores and the average relevance score used to select TP53 and PTEN for drug-sensitivity and molecular docking analyses. Figure S1: Comparison of four random survival forest models for biochemical recurrence prediction in the validation cohort. Figure S2: AlphaFold3 receptor-based molecular docking of PTEN- and TP53-associated candidate drugs.
