# Supplementary material for: Interpretable Machine Learning Prognostic Model Integrating Inflammatory-Nutritional Biomarkers for Post-Prostatectomy Biochemical Recurrence: Development, Internal Validation, and Translational Target Exploration
Source: Curr Oncol. 2026 Oct 9;33(10):608. doi: 10.3390/curroncol33100608 (PMC13703617; doi:10.3390/curroncol33100608)
Supplement: Supplementary file 1 [file curroncol-33-00608-s001.zip › Supplementary Figures S1-S2, Tables S1-S2.pdf]

Supplementary Materials

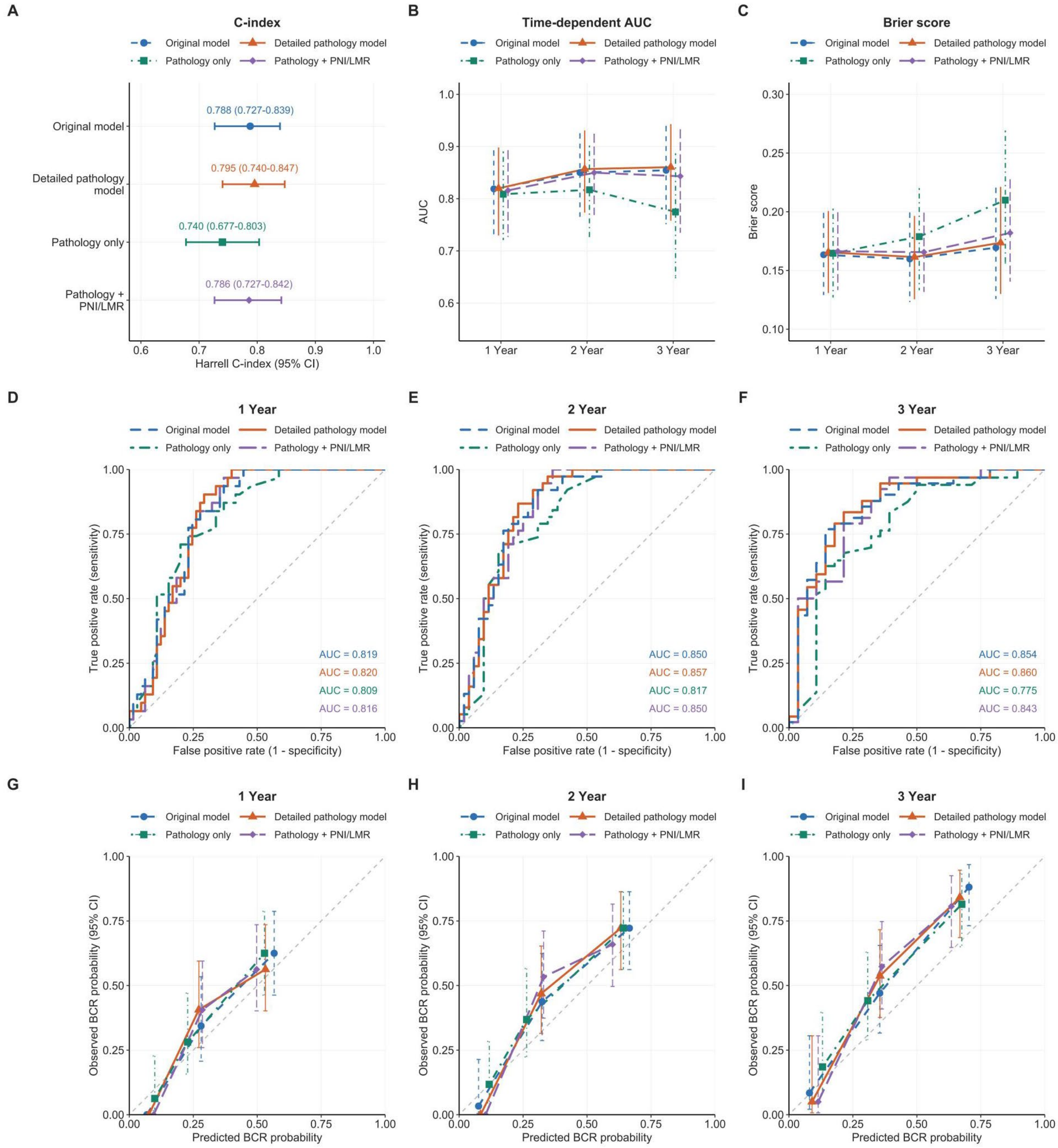

Figure S1. Comparison of four random survival forest models for biochemical recurrence prediction in the validation cohort. (A) C-index with 95% confidence intervals; (B) time-dependent AUC; (C) Brier score; (D–F) ROC curves at 1, 2 and 3 years; and (G–I) corresponding calibration plots. Models include the original model, detailed pathology model, pathology-only model, and the same pathology-only model with PNI and LMR added. Calibration error bars indicate available 95% confidence intervals for observed risk. PNI, prognostic nutritional index; LMR, lymphocyte-to-monocyte ratio; AUC, area under the curve.

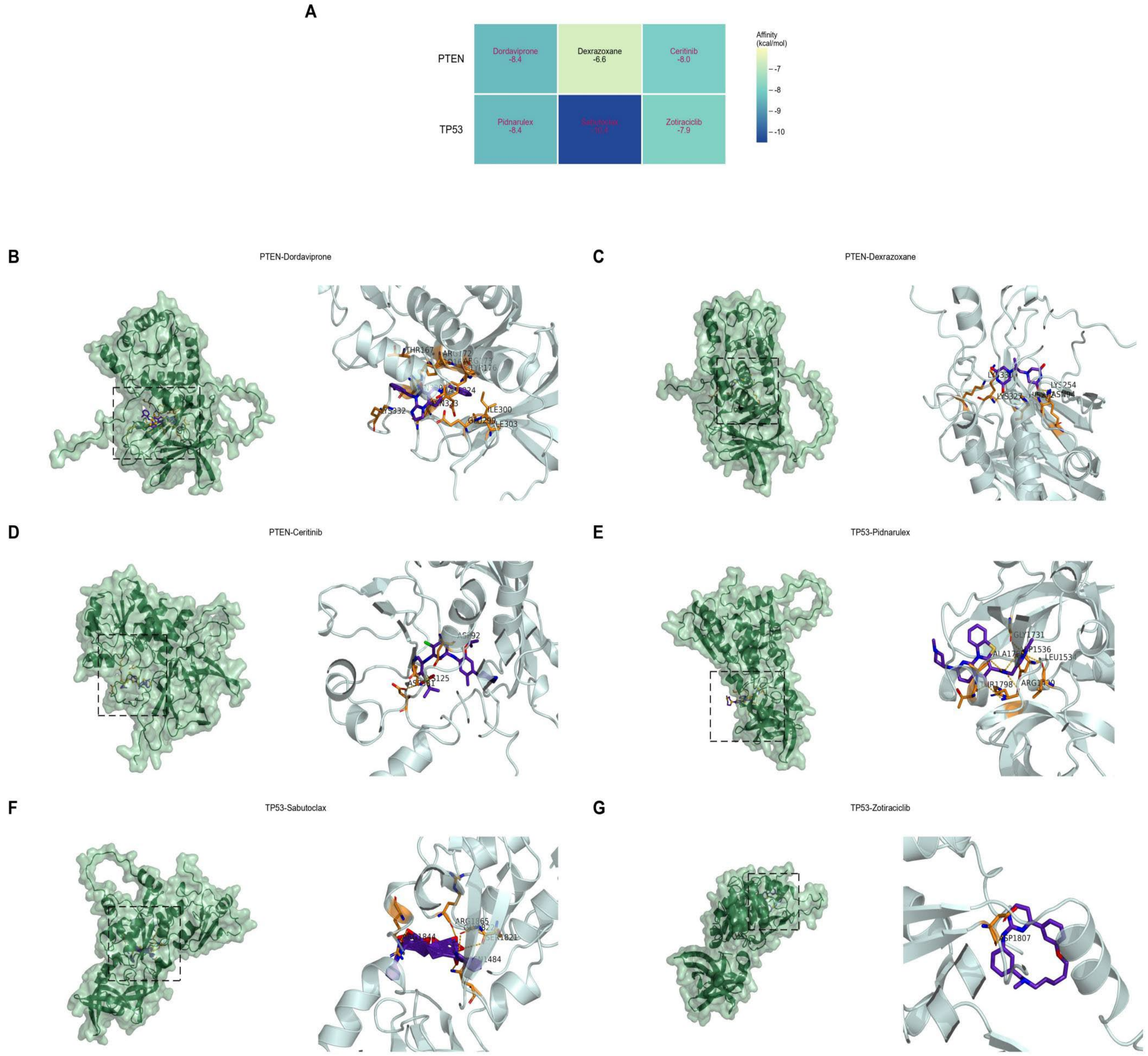

Figure S2. AlphaFold3 receptor-based molecular docking of PTEN- and TP53-associated candidate drugs. A, Heatmap of AutoDock Vina binding affinities obtained using AlphaFold3-predicted PTEN and TP53 receptor structures. B-G, PyMOL-based global receptor-ligand views and local polar-contact views for PTEN-Dordaviprone, PTEN-Dexrazoxane, PTEN-Ceritinib, TP53-Pidnarulex, TP53-Sabutoclax and TP53-Zotiraciclib.

Supplementary Table S1. Correlation analysis of patients with PIV<221 and PIV≥221 with clinicopathological features

Characteristics	PIV<221(*n*=176)	PIV≥221(*n*=144)	t/Z/chi-square	P value
Age	68.53 ± 6.78	69.33 ± 6.53	1.069	0.286
BMI [M(Q1, Q3)], kg/m²	24.91(23.17, 26.83)	25.58(24.08, 27.68)	1.991	0.047*
D'Amico risk classification (n, %)			8.517	0.014*
Low risk	29(16.48)	20(13.89)		
Intermediate risk	70(39.77)	38(26.39)		
High risk	77(43.75)	86(59.72)		
Postoperative ISUP Grade Group (n, %)			4.381	0.357
GG1 (Gleason ≤6)	21(11.93)	13(9.03)		
GG2 (Gleason 3+4)	54(30.68)	37(25.69)		
GG3 (Gleason 4+3)	46(26.14)	33(22.92)		
GG4 (Gleason 8)	19(10.80)	22(15.28)		
GG5 (Gleason 9-10)	36(20.45)	39(27.08)		
Surgical margin (n, %)			11.937	<0.001*
Negative	90(51.14)	46(31.94)		
Positive	86(48.86)	98(68.06)		
Perineural invasion (n, %)			1.314	0.252
No	41(23.30)	26(18.06)		
Yes	135(76.70)	118(81.94)		
Lymphovascular invasion (n, %)			3.888	0.049*
No	137(77.84)	98(68.06)		
Yes	39(22.16)	46(31.94)		
Pathological lymph node status (n, %)			0.168	0.919
pN0	110(62.50)	93(64.58)		
pN1	11(6.25)	8(5.56)		
pNx	55(31.25)	43(29.86)		
Pathological T stage (n, %)			1.684	0.640
pT2	109(61.93)	89(61.81)		
pT3a	24(13.64)	19(13.19)		
pT3b	32(18.18)	31(21.53)		
pT4	11(6.25)	5(3.47)		

**means the p-value<0.05 is considered statistically significant; BMI, body mass index; PIV, pan-immune-inflammation value. ISUP, International Society of Urological Pathology; GG, Grade Group; pT, pathological T stage.*

Supplementary Table S2. Correlation analysis of patients with LB-PSAD<0.395 and LB-PSAD≥0.395 with clinicopathological features

Characteristics	LB-PSAD<0.395(*n*=219)	LB-PSAD≥0.395(*n*=101)	t/Z/chi-square	P value
Age	68.63 ± 6.53	69.44 ± 6.97	0.974	0.331
BMI [M(Q1, Q3)], kg/m²	25.31(23.58, 27.22)	25.25(23.51, 27.24)	0.094	0.925
D'Amico risk classification (n, %)			54.334	<0.001*
Low risk	48(21.92)	1(0.99)		
Intermediate risk	89(40.64)	19(18.81)		
High risk	82(37.44)	81(80.20)		
Postoperative ISUP Grade Group (n, %)			26.339	<0.001*
GG1 (Gleason ≤6)	33(15.07)	1(0.99)		
GG2 (Gleason 3+4)	70(31.96)	21(20.79)		
GG3 (Gleason 4+3)	51(23.29)	28(27.72)		
GG4 (Gleason 8)	26(11.87)	15(14.85)		
GG5 (Gleason 9-10)	39(17.81)	36(35.64)		
Surgical margin (n, %)			8.419	0.004*
Negative	105(47.95)	31(30.69)		
Positive	114(52.05)	70(69.31)		
Perineural invasion (n, %)			10.859	<0.001*
No	57(26.03)	10(9.90)		
Yes	162(73.97)	91(90.10)		
Lymphovascular invasion (n, %)			12.867	<0.001*
No	174(79.45)	61(60.40)		
Yes	45(20.55)	40(39.60)		
Pathological lymph node status (n, %)			8.453	0.015*
pN0	137(62.56)	66(65.35)		
pN1	8(3.65)	11(10.89)		
pNx	74(33.79)	24(23.76)		
Pathological T stage (n, %)			18.177	<0.001*
pT2	151(68.95)	47(46.53)		
pT3a	27(12.33)	16(15.84)		
pT3b	35(15.98)	28(27.72)		
pT4	6(2.74)	10(9.90)		

**means the p-value<0.05 is considered statistically significant; BMI, body mass index; PNI, perineural invasion; LMR, lymphocyte-to-monocyte ratio; PIV, pan-immune-inflammation value; LB-PSAD, lymphocyte-based prostate-specific antigen density; ISUP, International Society of Urological Pathology; GG, Grade Group; pT, pathological T stage.*
